# A randomised controlled trial of mentalization-based treatment versus structured clinical management for patients with comorbid borderline personality disorder and antisocial personality disorder

**DOI:** 10.1186/s12888-016-1000-9

**Published:** 2016-08-30

**Authors:** Anthony Bateman, Jennifer O’Connell, Nicolas Lorenzini, Tessa Gardner, Peter Fonagy

**Affiliations:** 1 University College London, London, UK; 2 The Anna Freud Centre, London, UK; 3 Research Department of Clinical, Educational and Health Psychology, University College London, London, UK

**Keywords:** Borderline personality disorder, Antisocial personality disorder, Randomised controlled trial, Mentalization-based treatment, Aggression, Anger, Treatment outcome

## Abstract

**Background:**

Antisocial personality disorder (ASPD) is an under-researched mental disorder. Systematic reviews and policy documents identify ASPD as a priority area for further treatment research because of the scarcity of available evidence to guide clinicians and policymakers; no intervention has been established as the treatment of choice for this disorder. Mentalization-based treatment (MBT) is a psychotherapeutic treatment which specifically targets the ability to recognise and understand the mental states of oneself and others, an ability shown to be compromised in people with ASPD. The aim of the study discussed in this paper is to investigate whether MBT can be an effective treatment for alleviating symptoms of ASPD.

**Methods:**

This paper reports on a sub-sample of patients from a randomised controlled trial of individuals recruited for treatment of suicidality, self-harm, and borderline personality disorder. The study investigates whether outpatients with comorbid borderline personality disorder and ASPD receiving MBT were more likely to show improvements in symptoms related to aggression than those offered a structured protocol of similar intensity but excluding MBT components.

**Results:**

The study found benefits from MBT for ASPD-associated behaviours in patients with comorbid BPD and ASPD, including the reduction of anger, hostility, paranoia, and frequency of self-harm and suicide attempts, as well as the improvement of negative mood, general psychiatric symptoms, interpersonal problems, and social adjustment.

**Conclusions:**

MBT appears to be a potential treatment of consideration for ASPD in terms of relatively high level of acceptability and promising treatment effects.

**Trial registration:**

ISRCTN ISRCTN27660668, Retrospectively registered 21 October 2008

## Background

Borderline personality disorder (BPD) and antisocial personality disorder (ASPD) are both disorders with high levels of comorbid psychiatric illness. The most frequent comorbid psychiatric disorders in BPD are anxiety and affective disorders, with lifetime prevalence for these at approximately 85 %, followed by substance use disorders at approximately 79 % [1–4]. Co-existence of other psychiatric disorders in BPD has been reported as 41–83 % for major depression, 12–39 % for dysthymia [5], and 39 % for narcissistic personality disorder [6, 7]. Regarding antisocial personality disorder (ASPD), over 90 % of those with the condition have at least one other psychiatric disorder [8], at least 50 % have co-occurring anxiety disorders [9] and 25 % have a depressive disorder [10]. Notwithstanding the varying current views in relation to the classification of categories of personality disorder [11, 12], individuals who meet criteria for both ASPD and BPD can be considered as showing a particularly complex and severe form of personality disorder in so far as they are likely to present with particularly high levels of both DSM Axis I and Axis II comorbidity [13]. Amongst the general (UK) population, the prevalence of individuals meeting both BPD and ASPD diagnostic criteria is low (0.3 %) [14], but is increased in forensic samples with a higher degree of presumed dangerousness; it has been found to be significantly associated with a greater degree of violence [15, 16].

There is sufficient overlap between ASPD and BPD that considering them as separate disorder entities may seem unwarranted [11]. Yet, while future classifications may do away with the distinction [17], or at least substantially modify it (as implied by Section III of DSM-5), current nosology incorporates the difference notwithstanding similarities in symptomatology [18] and trait domains (namely, antagonism and disinhibition) [19]. In particular, crossover includes marked impulsivity and unpredictability, difficulties with emotional regulation and controlling anger, disregard for safety of self, and behaviour that can be considered by others to appear manipulative (for those with BPD, such behaviour is conducted with the intention of eliciting care and concern from others; for those with ASPD, such behaviour is conducted with the intention of gaining personal profit and power over others) [20–22]. Nonetheless, it is easy to see why the two disorders may be considered as distinctly different from each other. The key, frequently-noted differences include the following: those diagnosed with ASPD tend to have an inflated self-image, whilst those diagnosed with BPD tend to have a negative and devalued self-image; those diagnosed with ASPD pose more of a risk to others due to their tendency towards interpersonal violence, whilst those diagnosed with BPD pose more of a risk to themselves due to their tendency to self-damaging and self-destructive behaviours; those diagnosed with ASPD tend to lack empathy and be indifferent to or contemptuous of the feelings and sufferings of others, whilst those diagnosed with BPD are likely to display reduced cognitive empathy but enhanced affective empathy (the so-called “borderline empathy paradox”) [23]. In reality, although the prototypic presentations of the two diagnoses appear to be at variance and may be almost polar opposites, the prominent symptoms appear across diagnostic groups as well as varying across time—both within and between individuals. A recent study of nearly 1,000 inpatients found a general personality disorder factor that underlies all personality disorder diagnoses [18]. Bi-factor analyses of the DSM personality disorder criteria indicated that they load on to a general factor that includes all the BPD criteria, rather than the latter representing a separate personality disorder category. It appears that BPD might be better understood as being at the core of personality pathology more generally, rather than as a type of personality disorder. This approach helps to make sense of the high levels of comorbidity with other personality disorders, including ASPD, found in BPD patients.

In line with this assumption, we suggest that there may be a common element of failures in social cognition associated with both personality disorders [24]; in particular they share deficits and distortions of mentalization (the process of making sense of the self and of others in terms of mental states—e.g. beliefs, thoughts, feelings, desires). It appears that those with BPD tend to mentalize ‘normally’ except in the context of attachment relationships, in which emotional arousal occludes the ability to accurately interpret mind states (both their own minds and those of others)—particularly when the fear of real or imagined abandonment arises. Difficulties with mentalization manifest slightly differently for antisocial individuals, who show a more general and deeper impairment, including deficits in the recognition of basic emotions [25], and perform far worse than controls on subtle tests of mentalizing [26, 27]. Deficits in social cognition in general and the capacity to link mental states to behaviour in particular are commonly identified in association with antisocial behaviour [28, 29]. Some (but not all [30]) individuals with ASPD show a blend of perspective-taking problems and difficulty in reading others’ mental states [31–37]; this is consistent with the mentalization literature’s deficit theory as well as other theories of antisocial behaviour [38, 39]. It has been suggested that one pathway to adult antisocial personality leads from early child conduct disorder via alcohol abuse in early adolescence to compromised function (and maturational delay) of the cognitive control system, of which mentalization is a part [40, 41], and which matures throughout adolescence and into early adulthood. Hence, deficits in the ability to mentalize could reflect compromised (i.e. delayed) development of the cognitive control system, brought about by early substance misuse.

Mentalization-Based Treatment—a manualised psychotherapeutic intervention which specifically focuses on improving the capacity to mentalize—has been shown to be effective for improving clinically significant and self-reported outcomes for patients with BPD, including reducing frequency of suicide, severe self-harm, and hospital admission as well as improving general symptomatology and social and interpersonal functioning [42]. While the presence of comorbid Axis II diagnoses appears to have a negative impact on outcomes for BPD patients undergoing standard clinical management, there has been preliminary work to suggest that MBT may be more beneficial for patients whose BPD is embedded in other Axis II personality disturbances, including that of ASPD [43]. The authors propose that the mentalization model can also be applied to ASPD and that MBT may be effective for addressing symptoms of ASPD as well as of BPD.

The mentalization model of antisocial behaviour is developmental, premised on the dysfunction of the attachment system that then temporarily inhibits affect regulation and mentalizing abilities [44–47]. Antisocial behaviour and violence tend to occur when an understanding of others’ mental states is developmentally compromised (fragile) and prone to being lost when the attachment system is activated by perceived threats to self-esteem, such as interpersonal rejection or disrespect [48]. Normally, mentalizing (i.e. envisioning the subjective state of the victim) precludes violence [49]; this means that individuals with vulnerable mentalizing capacities can be behaviourally volatile in moments of interpersonal stress. Supporting the capacity to identify others’ emotions and intentions may not only assist social functioning but also reduce the risk of antisocial behaviour. Indeed, mentalizing has been shown to be a protective factor against aggression in people with violent traits [32], encouraging mentalizing has also been shown to reduce school violence [50, 51], and studies of forensic patients with personality disorder have found that participants’ views of the processes by which therapeutic change occurred identified realisations that reflected improved mentalizing [52, 53].

This study tests the hypothesis that patients with comorbid BPD and ASPD receiving outpatient MBT would be more likely to show improvements in symptoms related to aggression than those offered an outpatient structured protocol of similar intensity but excluding MBT components. This comorbid population was selected for pragmatic reasons, as a subsample of a trial originally designed to compare MBT to Structured Clinical Management (SCM) in a sample of consecutive referrals to a personality disorder unit that specialises in BPD [49]. At the time of the original trial, MBT had not yet been indicated for ASPD and the trial was not designed with this diagnosis in mind. Nevertheless, our original design allows us to measure change in important psychological features directly related to characteristics of ASPD such as anger, hostility, impulsivity (as reflected in self-harm and suicide) and difficulty in relaxing interpersonal control related to loss of dignity, self-worth and self-respect [54]. Mood disorders, particularly anxiety and depression, are known to co-occur with ASPD [9, 55, 56] and to be frequent triggers for aggression [57, 58]. The trial design also enabled us to test whether MBT may be effective in reducing negative affect states, particularly depression and anxiety. Finally, the study design enabled us to measure change due to treatment in common consequences of aggression, such as psychiatric symptoms including quality of familial and social relationships, and subjective wellbeing.

## Method

### Design

This subgroup analysis was part of a larger pragmatic randomised superiority trial that investigated MBT as a treatment for patients with BPD in an outpatient context by non-specialist mental health practitioners at a publicly funded clinical service [42]. To control for the non-specific benefits of a structured treatment, the comparison group also received a protocol-driven therapy, SCM, in an outpatient context representing best current clinical practice [59]. Practitioners in both arms received the same level of training and equivalent supervision.

### Participants

Participants were recruited from consecutive referrals for personality disorder treatment from clinical services between January 2003 and February 2006. All participants were assessed using the Structured Clinical Interview for DSM-IV (SCID-I and SCID-II) [60, 61]. Inclusion criteria were (1) diagnosis of ASPD and comorbid BPD; (2) suicide attempt or episode of life-threatening self-harm within the past 6 months; and (3) age 18–65. Exclusion criteria were kept to a minimum. Patients were excluded if they currently (1) were in long-term psychotherapeutic treatment; (2) met DSM-IV criteria for psychotic disorder or bipolar I disorder; (3) had opiate dependence requiring specialist treatment; or (4) had mental impairment or evidence of organic brain disorder. Current psychiatric inpatient treatment, temporary residence, drug/alcohol misuse and comorbid personality disorder were not exclusion criteria.

One hundred and fifty-eight patients attended for interview. Of these, five did not have BPD, 94 did not have ASPD, two had opiate dependence, one had bipolar I disorder, one had a psychotic disorder, and three could not be contacted after the diagnostic interview. Of the 52 patients enrolled, 12 refused randomisation, leaving 40 with a dual diagnosis of ASPD and BPD, who were randomised to one of the two outpatient treatment programmes (MBT = 21, SCM = 19). Seventy-five percent of the MBT group were male, with a mean age of 31.50 (SD = 8.20) years; 8 % were employed and 76 % were receiving welfare benefits at the time. They had a mean number of 3.1 (SD = 1.3) Axis I and 3.3 (SD = 0.9) Axis II diagnoses. Of the SCM group, 62 % were males, with a mean age of 30.00 (SD = 7.10) years; 6 % were employed and 94 % were receiving benefits. They had a mean number of 3.0 (SD = 1.5) Axis I and 2.9 (SD = 1.0) Axis II diagnoses. None of the participants had ever been married^1^. We tested the relative severity of the groups in terms of the number of Axis I and II diagnoses and found no differences on the Kruskal-Wallis test on either of these variables (χ ^2^ = 0.05, df = 1, *p =* .84, and χ ^2^ = 0.66, df = 1, *p =* .41 for number of Axis I and Axis II diagnoses respectively).

### Procedure

Patients referred to St Ann’s Hospital’s specialist personality disorder service were randomly assigned to one of two active treatment arms and assessed at entry and over the course of an 18-month treatment at 6, 12, and 18 months. The study was approved by Barnet, Enfield and Haringey Local Research and Ethics Committee (ref: 658) and conducted at the Halliwick personality disorder service and in a community outpatient facility. Patients were provided with written information and consented only after complete description of the study. Randomisation followed consent, enrolment, and baseline assessment by a research assistant at St Ann’s Hospital. Treatment allocation was made offsite via telephone randomisation using a stochastic minimisation program (MINIM) balancing for age (blocked as 18–25, 26–30, >30 years), gender, and presence of ASPD. A study psychiatrist informed patients of their allocation. All treatments were funded by the NHS. Participants were not paid.

Both treatments were conducted within a structured framework following principles outlined elsewhere [62] and summarised in NICE guidance for BPD [21]. This included crisis contact and crisis plans, pharmacotherapy, general psychiatric review, and written information about treatment. Crisis plans were developed collaboratively within each treatment team for all patients. MBT therapists focused on helping patients reinstate mentalizing during a crisis via telephone contact. SCM therapists focused on support and problem solving. Efforts were made to keep all patients in treatment if they missed appointments. Both therapies were offered weekly to ensure equivalent therapeutic intensity in both groups. All patients were offered approximately 140 sessions of therapy in total. Seventy-five percent across the two groups met criteria for completers - at least 70 sessions attended over the first year. There was no difference in the distribution of completer categories across the groups (χ ^2^ = 1.87, df = 2, *p =* .18). We also tested the amount of treatment received by each group in terms of individual, group and total sessions and there were no differences on the Kruskall-Wallis test on any of these variables (χ ^2^ = 0.86, df = 1, *p =* .38, χ ^2^ = 1.51, df = 1, *p =* .22, χ ^2^ = 1.80, df = 1, *p =* .17 for individual, group and total sessions respectively).

#### Mentalization-based treatment

MBT is a manualised, structured treatment that integrates cognitive, psychodynamic and relational components of therapy, with a basis in attachment theory and a rigorous focus on improving mentalizing (the ability to understand the mental states of oneself and others). It consisted of 18 months of weekly combined individual and group psychotherapy provided by two different therapists [63].

#### Structured clinical management

An outpatient SCM protocol was developed through the Barnet, Enfield and Haringey Mental Health NHS Trust to reflect best generic practice for personality disorder offered by non-specialist practitioners within UK psychiatric services. Weekly individual and group sessions were offered, with appointments every 3 months for psychiatric review. Therapy was based on a counselling model closest to a supportive approach with case management, advocacy support, and problem-oriented psychotherapeutic interventions; the treatment model has been manualised [59].

#### Medication

Patients were prescribed medication by a member of the treatment team (MBT condition) or their consultant psychiatrist (SCM condition). All patients were offered medication reviews every 3 months. All prescribers were asked to adhere to APA guidelines [64]. Prescribing patterns and dosage were monitored by review of medical records every 6 months.

### Outcomes

Outcomes were classified according to their relation to aggression. Anger, domineering relationships, hostility, paranoid ideation and signs of impulsivity (suicidality and self-harm) were the target outcomes as indicators of problematic aggression. Anxiety, depression, and a summary indicator of psychiatric symptomatic distress were assessed as possible drivers of overt aggression. Lastly, we addressed consequences secondary to problematic aggression: problems in interpersonal relationships, social, familial and global functioning.

### Measures

Clinician-rated anger was assessed at the beginning and end of treatment by assessors blind to treatment group using SCID-II questions related to item 4 (‘irritability and aggressiveness as indicated by repeated physical fights and assaults’) of the diagnostic criteria for ASPD. Patients’ subjective experience of hostility, paranoid ideation and general symptom distress was measured using the Symptom Checklist-90—Revised (SCL-90-R) [65], and depression was assessed by using the Beck Depression Inventory (BDI) [66]. The hostility subscale of the SCL-90 consists of 6 items (items 11, 24, 63, 67, 74, 81) measuring feelings of annoyance and irritability and urges to hurt others as well as actions such as breaking things, getting into arguments, and shouting at others or throwing things. The paranoid ideation subscale is also 6 items, identifying feelings that others are to blame and unappreciative along with a general sense of distrust in others and the world. Self-harm and suicidality were assessed at interview and confirmed from medical records. Social adjustment and interpersonal functioning were measured using the modified Social Adjustment Scale (SAS)–self-report [67], and the Inventory of Interpersonal Problems (IIP)–circumplex version [68, 69], which provided a scale of domineering as well as general interpersonal problems. The instruments provide an assessment of an individual’s work, spare time activities, and family life, as well as difficulties with interpersonal functioning. Hospital admission statistics were based on local computerised medical records. Global functioning was rated at the beginning and end of treatment using the Global Assessment of Functioning (GAF), which has been found to show less improvement than diagnostic symptoms in studies of a naturalistic follow-on design [70]. Assessors were blind to treatment group, and their assessment was compared with clinician ratings to indicate reliability.

### Analysis plan

Treatment group differences were assessed with *t* tests for independent samples. Change in continuous variables was analysed with mixed effects linear regressions as function of group treatment and time. Incidence of suicidal and severe self-injurious behaviours and episodes of hospitali*s*ation, assessed in 6-month periods both in terms of counts and as dichotomous data (present or absent in each period), was used to indicate the severity of life-threatening parasuicidal behaviour taken at baseline for the 6 months prior to treatment and for each 6-month period until the end of treatment at 18 months. Count data were analysed using a mixed effects Poisson regression model; for binary data a mixed effects logistic regression model was used.

## Results

### Target symptoms

Table 1 shows means and standard deviations of anger, domineering interpersonal style as measured by the IIP, and hostility as assessed on the SCL-90 at all time points. Anger, measured by the presence of anger-related criteria on the SCID-II ASPD module, was collected at baseline and at the end of the 18 months of treatment. Both the MBT and SCM groups presented with similar levels of anger at the beginning of treatment, but differed significantly by 18 months (*t* = 2.05, *p* < 0.05). Mixed effects regression revealed significant difference in the linear change observed. Neither the SCM nor the MBT group showed significant changes in domineering interpersonal style as measured by the IIP. Self-rated hostility, however, decreased in both groups. While linear decline was not statistically significantly steeper, both observed and model-predicted hostility was significantly lower in the MBT than the SCM group. Paranoia symptoms, measured by the SCL-90, and paranoid ideation, measured by the SCID-II, showed significantly more improvement in the MBT than the SCM group, and at 18 months, paranoia and paranoid ideation scores were significantly lower.

**Table 1:** Changes in anger (SCID-II), domineering interpersonal problems (IIP), Hostility (SCL-90) and paranoia (DSM and SCL-90)

	Anger (DSM)					Domineering (IIP)					Hostility (SCL-90)				
	Unadjusted observed mean (SD)					Unadjusted observed mean (SD)					Unadjusted observed mean (SD)				
	MBT		SCM		*t*	MBT (*n* = 21)		SCM (*n* = 19)		*t*	MBT (*n =* 21)		SCM (*n =* 19)		*t*
Baseline	2.76 (*n =* 21)	(0.62)	2.68 (*n =* 19)	(0.75)	-0.36	-0.12	(0.77)	-0.33	(0.40)	-0.10	1.73	(1.20)	2.04	(1.04)	0.88
6 months						-0.16	(0.73)	-0.16	(0.45)	-0.02	1.67	(1.08)	1.86	(0.87)	0.61
12 months						-0.18	(0.69)	-0.23	(0.41)	-0.30	1.43	(0.96)	1.88	(0.78)	1.60
18 months	1.25 (*n =* 8)	(0.46)	2.00 (*n =* 8)	(0.93)	2.05*	-0.22	(0.43)	-0.06	(0.46)	1.06	0.85	(0.60)	1.57	(0.69)	3.53***
	Adjusted model coefficients (95 % CI)^a^					Adjusted model coefficients (95 % CI)^b^					Adjusted model coefficients (95 % CI)^b^				
Model: Wald *χ* ^2^	35.56*** (df = 3)^a^					3.51 (df = 4)					54.83*** (df = 5)				
Linear change (both groups)	-0.23*		(-0.41, -0.04)			0.07		(-0.10, 0.24)			-0.48**		(-0.84, -0.12)		
Quadratic change (both groups)						0.00		(-0.05, 0.05)			-0.17*		(-0.32, -0.02)		
Differential linear change (MBT)	-0.28*		(-0.54, -0.02))			-0.11		(-0.22, 0.05)			-0.27		(-0.54, 0.01)		
Group differences at 18 months	-0.75*		(-1.41, -0.09)			-0.13		(-0.41, 0.16)			-0.60**		(-1.02, -0.18)		
	Paranoia (SCL-90)					Paranoid ideation (DSM)									
	Unadjusted observed mean (SD)					Unadjusted observed mean (SD)									
	MBT (*n* = 21)		SCM (*n* = 19)		*t*	MBT		SCM		*t*					
Baseline	1.86	(1.03)	1.94	(0.72)	0.29	2.14 (*n =* 21)	(0.91)	2.05 (*n =* 19)	(0.97)	-0.30					
6 months	1.62	(0.99)	2.00	(0.80)	1.24										
12 months	1.52	(1.00)	1.81	(0.78)	1.02										
18 months	0.85	(0.57)	1.47	(0.70)	3.06**	1.00 (*n =* 8)	(0.00)	1.88 (*n =* 8)	(0.99)	2.50**					
	Adjusted model coefficients (95 % CI)^c^					Adjusted model coefficients (95 % CI)^d^									
Model: Wald *χ* ^2^	22.77*** (df = 3)					22.77*** (df = 3)									
Linear change (both groups)	-0.12		(-0.29, -0.05)			-0.12		(-.029, 0.05)							
Differential linear change (MBT)	-0.29*		(-0.53, -0.36)			-0.28*		(-0.53, –0.04)							
Group differences at 18 months	-0.75*		(-1.51, -0.01)			-0.75*		(-1.51, 0.01)							

*MBT* Mentalization-Based Treatment, *SCM* Structured Clinical Management, *t t*-test for independent samples, *χ* ^2^ chi-squared, *CI* confidence interval, *df* degrees of freedom

*p < 0.05; **p < 0.01; ***p < 0.001

^a^fixed-effects linear regression

^b^mixed-effects linear regression with random intercept

^c^mixed-effects linear regression with random intercept

^d^mixed-effects linear regression with random slope

Occurrence of suicide attempts, episodes of self-harm and hospital admissions during the last 6 months were registered and counted, and summary scores are displayed in Table 2. A composite variable was created to count the number of patients free of any of these three severe clinical events in the last 6 months. Mixed effects logistic regression with random intercept confirm the significant observed end of treatment group differences (62 % vs 21 %). Although the number of patients attempting suicide was not significantly different in the logistic regression model, the number of suicide attempts modelled using mixed effects Poisson regression suggested significant superiority of MBT over SCM. For self-harm, both the mean number of self-harm attempts and the number of those who self-harmed suggested greater benefit for participants in the MBT group. Similarly, numbers of hospital admissions were lower in this group. At 18 months, both model-estimated (intention-to-treat) and observed (per protocol) self-harm frequency were significantly lower for the MBT group. Model-estimated (mixed-effects Poisson) frequency of hospital admissions was slightly lower for the MBT group at 18 months.

**Table 2:** Changes in impulsive acts (suicidality and self-harm)

	Patients without severe clinical events					Patients who presented suicide attempts					Number of suicide attempts				
	n/N(%)					n/N(%)					Unadjusted observed mean (SD)				
	MBT		SCM		*χ* ^2^	MBT		SCM		*χ* ^2^	MBT (*n =* 21)		SCM (*n =* 19)		*t*
Baseline	0/21	0.00 %	0/19	0.00 %		19/21	90.48 %	15/19	78.95 %	1.04	1.38	(1.07)	1.32	(0.95)	-0.20
6 months	2/21	9.52 %	1/19	5.26 %	0.26	11/21	52.38 %	11/19	57.89 %	0.12	0.57	(0.60)	0.89	(0.99)	1.26
12 months	6/21	28.57 %	2/19	10.53 %	2.03	8/21	38.10 %	10/19	52.63 %	0.85	0.43	(0.60)	0.79	(1.03)	1.37
18 months	13/21	61.90 %	4/19	21.05 %	6.81**	0/21	0.00 %	4/19	21.05 %	4.91*	0.00	(0.00)	0.37	(0.83)	2.04*
	Modelled odds ratios (95 % CI) ^a^					Modelled odd ratios (95 % CI) ^a^					Adjusted model coefficients (95 % CI)^b^				
Model: Wald *χ* ^2^	12.48** (df = 3)					23.67*** (df = 3)					36.29***				
Linear change (both groups)	8.42*		(1.18, 60.15)			0.20**		(0.08, 0.51)			0.70**		(0.55, 0.88)		
Differential linear change (MBT)	2.83		(0.32, 24.79)			0.34		(0.10, 1.16)			0.61*		(0.41, 0.91)		
Group differences at 18 months	61.45*		(1.17, 60.15)			0.09		(0.01, 1.35)			0.25**		(0.09, 0.71)		
	Patients who self-harmed					Number of self-harm episodes					Number of hospital admissions				
	n/N(%)					Unadjusted observed mean (SD)					Unadjusted observed means (SD)				
	MBT		SCM		*χ* ^2^	MBT (*n =* 21)		SCM (*n =* 19)		*t*	MBT (*n =* 21)		SCM (*n =* 19)		*t*
Baseline	16/21	76.19 %	14/19	73.68 %	0.03	5.90	(6.69)	3.79	(3.61)	-1.23	0.57	(0.60)	0.32	(0.58)	-1.37
6 months	17/21	80.95 %	17/19	89.47 %	0.57	3.14	(3.02)	3.11	(3.57)	-0.04	0.24	(0.44)	0.21	(0.42)	-0.20
12 months	10/21	47.62 %	15/19	78.95 %	4.18*	2.14	(2.87)	2.42	(2.39)	0.37	0.10	(0.30)	0.32	(0.75)	1.24
18 months	8/21	38.10 %	13/19	68.42 %	3.68	0.62	(0.97)	2.63	(3.65)	2.43*	0.00	(0.00)	0.16	(0.37)	1.93*
	Modelled odds ratios (95 % CI)^a^					Adjusted model coefficients (95 % CI)^b^					Adjusted model coefficients (95 % CI) ^b^				
Model: Wald *χ* ^2^	13.34** (df = 3)					98.73*** (df = 4)					17.38** (df = 4)				
Linear change (both groups)	0.72		(0.35, 1.49)			-0.12		(-0.27, 0.35)			0.89		(0.46, 1.74)		
Differential linear change (MBT)	0.30*		(0.10, 0.91)			-0.29**		(-0.50, -0.08)			0.28		(0.06, 1.27)		
Group differences at 18 months	0.03*		(0.01, 0.63)			-0.66***		(-0.98, -0.34)			0.06*		(0.00, 0.81)		

*MBT* Mentalization-Based Treatment, *SCM* Structured Clinical Management, *t t*-test for independent samples, *χ* ^2^ chi-squared, *CI* confidence interval; df, degrees of freedom

*p < 0.05; **p < 0.01; ***p < 0.001

^a^mixed-effects logistic regression with random intercept

^b^mixed-effects Poisson regression with random intercept

### Drivers of aggression

Anxiety and depression, as measured by the SCL-90 and the BDI, were both seen to improve significantly across both groups during treatment, following a linear pattern for depression, and both a linear and a quadratic pattern for anxiety, suggesting a fast reduction in anxiety during the first months of treatment. Table 3 shows the observed means for these two scores ordered by treatment group and follow-up time point. At the 18-month point, both anxiety and depression scores (observed and predicted by mixed-effects models) were significantly lower for the MBT group than for the SCM group. The combined symptom distress scores (Global Severity Index; GSI) similarly suggested greater ultimate benefit from MBT, although the mixed effects regression did not indicate significantly more rapid (steeper linear change) improvement.

**Table 3:** Changes in mood (anxiety and depression) and general symptom distress (GSI)

	Anxiety (SCL-90)					Depression (BDI)					GSI (SCL-90)				
	Unadjusted observed mean (SD)					Unadjusted observed mean (SD)					Unadjusted observed mean (SD)				
	MBT (*n =* 21)		SCM (*n =* 19)		*t*	MBT (*n =* 21)		SCM (*n =* 19)		*t*	MBT (*n =* 21)		SCM (*n =* 19)		*t*
Baseline	1.83	(0.91)	2.18	(0.82)	1.30	27.51	(11.42)	30.58	(7.51)	0.99	1.75	(0.74)	2.01	(0.55)	1.25
6 months	1.83	(0.85)	2.28	(0.89)	1.62	24.68	(10.45)	29.00	(8.06)	1.45	1.73	(0.80)	2.03	(0.67)	1.29
12 months	1.59	(1.02)	2.05	(0.80)	1.61	19.19	(11.18)	26.56	(8.71)	2.31**	1.52	(0.87)	2.00	(0.66)	1.91*
18 months	1.00	(0.67)	1.62	(0.75)	2.74**	13.14	(7.25)	21.99	(9.20)	3.39***	0.96	(0.52)	1.57	(0.63)	3.37***
	Adjusted model coefficients (95 % CI)^a^					Adjusted model coefficients (95 % CI)					Adjusted model coefficients (95 % CI)				
Model: Wald *χ* ^2^	54.07*** (df = 4)					92.30*** (df = 3)^a^					87.16*** (df = 5)^b^				
Linear change (both groups)	-0.61***		(-0.86, -0.36)			-2.82***		(-4.05, -1.59)			-.060***		(-0.90, -0.29)		
Quadratic change (both groups)	-0.14***		(-0.21, -0.07)								-0.18**		(-0.32, -0.05)		
Differential linear change (MBT)	-0.08		(-0.24, -0.08)			-2.04*		(-4.74, -0.34)			-0.15		(-0.34, 0.04)		
Group differences at 18 months	-0.59*		(-1.07, -0.12)			-8.96***		(-14.27, -3.65)			-0.47**		(-0.79, -0.15)		

*MBT* Mentalization-Based Treatment, *SCM* Structured Clinical Management, *t t*-test for independent samples, *χ* ^2^ chi-squared, *CI* confidence interval, *df* degrees of freedom

*p < 0.05; **p < 0.01; ***p < 0.001

^a^mixed-effects linear regression with random intercept

^b^mixed-effects linear regression with random slope

### Secondary consequences of aggression

As indicated in Table 4, patients showed comparable total scores on the GAF, IIP and SAS measures at baseline. Mixed-effect models showed a significantly greater linear improvement with time on GAF, and greater linear reduction on IIP and SAS adjustment problems for the MBT group, although both groups improved substantially. However, patients who received MBT showed significantly more improvements on overall functioning, interpersonal problems, and social adjustment scores at the end of treatment, in comparison to patients who received SCM.

**Table 4:** Changes in global functioning (GAF), problems in interpersonal relationships (IIP) and social adjustment (SAS)

	GAF (DSM)					IIP total					SAS total				
	Unadjusted observed mean (SD)					Unadjusted observed mean (SD)					Unadjusted observed mean (SD)				
	MBT		SCM		*t*	MBT (*n =* 21)		SCM (*n =* 19)		*t*	MBT (*n =* 21)		SCM (*n* = 19)		*t*
Baseline	38.57 (*n =* 21)	(9.09)	39.74 (*n =* 19)	(8.38)	0.42	2.04	(0.62)	2.11	(0.41)	0.43	2.71	(0.41)	2.81	(0.41)	0.78
6 months						1.87	(0.58)	2.03	(0.40)	1.04	2.53	(0.62)	2.72	(0.49)	1.06
12 months						1.66	(0.51)	2.01	(0.56)	2.06*	2.05	(0.41)	2.63	(0.44)	4.31***
18 months	62.71 (*n =* 21)	(16.66)	51.39 (*n =* 18)	(12.06)	-2.39**	1.33	(0.48)	1.76	(0.44)	2.94**	1.69	(0.45)	2.27	(0.51)	3.78***
	Adjusted model coefficients (95 % CI)					Adjusted model coefficients (95 % CI)					Adjusted model coefficients (95 % CI)				
Model: Wald *χ* ^2^	107.57 (df = 3)^b^					57.47*** (df = 3)^b^					82.53*** (df = 4)^a^				
Linear change (both groups)	3.82***		(2.01, 5.63)			-0.11**		(-0.18, -0.04)			-0.22***		(-0.35, -0.10)		
Quadratic change (both groups)															
Differential linear change (MBT)	4.22**		(1.75, 6.70)			-0.13*		(-0.23, -0.03)			-0.19*		(-0.36 -0.02)		
Group differences at 18 months	11.51**		(4.13, 18.89)			-0.44**		(-0.73, -0.16)			-0.60***		(-0.88, -0.33)		

*MBT* Mentalization-Based Treatment, *SCM* Structured Clinical Management, *t t*-test for independent samples, *χ* ^2^ chi-squared, *CI* confidence interval, df: degrees of freedom

*p < 0.05; **p < 0.01; ***p < 0.001

^a^mixed-effects linear regression with random slope

^b^mixed-effects linear regression with random intercept

## Discussion

The aim of the study was to provide preliminary data to indicate whether MBT could reduce symptoms related to antisocial behaviour in patients with comorbid BPD and ASPD when compared with those offered an outpatient structured protocol of similar intensity, but excluding mentalizing components. The data collected is rare in its inclusivity of individuals with comorbid personality disorders, a key clinical group typically excluded from randomised controlled trials, and the results strongly suggest that MBT is an effective treatment for patients with BPD and comorbid ASPD. Patients treated with MBT showed a significantly greater reduction in the target symptoms of anger, hostility and paranoia than those in the SCM group. At the end of treatment, fewer patients in the MBT group presented with impulse control-related problems (suicidality and self-harm), and the frequency of suicide attempts and self-harm episodes was also significantly lower. Measures of negative mood and general psychiatric symptoms, which may identify a vulnerability to the aggressive acts that are a hallmark of ASPD, also showed significantly greater improvements and indicated better adjustment in the MBT group. Similarly, common sequelae of aggression—poor general functioning, interpersonal problems and social adjustment at the end of treatment—were improved following MBT in comparison with those who received SCM.

This study has a number of major limitations. A longer-term follow-up is required to ascertain whether differential effects are maintained; although a 5-year follow-up study is underway, as yet we have no data to report. The study is not adequately powered to demonstrate real differences between the two groups and we recognise that implications of the findings are restricted to the demonstration of feasibility rather than a demonstration of effectiveness [71]. The lack of specific outcome measures for indications of mentalizing ability (e.g. Reading the Mind in the Eyes Test, Movie for the Assessment of Social Cognition, Perspectives Taking Test, Reflective Functioning Questionnaire, Reflective Functioning Scale on the Adult Attachment Interview) is a major limitation that prevents us from gathering information on MBT’s purported mechanism of change. Additionally, indicators are required that are more central in the planning of services for ASPD than self-reported psychiatric symptoms and mental health diagnostic status after 18 months, including a measure of whether patients continue to meet criteria for formal diagnosis of ASPD. Furthermore, as this comorbid population was selected from a personality disorder unit that specialises in BPD for pragmatic reasons of availability, this study is significantly underpowered and unrepresentative of both the wider ASPD population and the settings in which they most commonly present.

Nevertheless, the results from this study demonstrate that randomisation and effective data collection are possible in a community setting from outpatients with ASPD. Further, we established that patients by and large adhere to the treatment protocol (dropout rates of 27 % for MBT and 26 % for SCM); additionally, qualitatively collected data (reported fully elsewhere [72]) strongly suggest that this patient group (perhaps surprisingly) value the intervention, and are particularly appreciative of the group format. MBT therefore appears to be a potential treatment of consideration for ASPD in terms of relatively high level of acceptability and promising treatment effects.

## Conclusions

In patients with comorbid ASPD and BPD, MBT is able to reduce anger, hostility, paranoia, and frequency of self-harm and suicide attempts, as well as improve negative mood, general psychiatric symptoms, interpersonal problems, and social adjustment. The authors recommend that MBT be further investigated in a significantly powered sample size of patients with a primary diagnosis of ASPD to establish its long-term effects on symptomatology specific to ASPD (e.g. aggression, violence, offending behaviour) as well as outcomes relevant to other Axis I and Axis II disorders (e.g. impulsiveness, social functioning, general wellbeing), whether MBT has an effect on diagnostic continuity for ASPD, and whether any changes can be attributed to change in mentalizing capacity.

## Abbreviations

ASPD: Antisocial personality disorder
BDI: Beck Depression Inventory
BPD: Borderline personality disorder
BSI: Brief Symptom Inventory
GAF: Global Assessment of Functioning
GSI: Global Severity Index
HCR-20: Historical, Clinical, Risk Management-20
IIP: Inventory of Interpersonal Problems
MBT: Mentalization-Based Treatment
OAS-M: Overt Aggression Scale – Modified
RCT: Randomised controlled trial
SAS: Social Adjustment Scale
SCID: Structured Clinical Interview for DSM-IV
SCL-90-R: Symptom Checklist-90 – Revised
SCM: Structured Clinical Management
SF-36: Short Form Health Survey

## Footnotes

1 For further demographic and clinical details of the sample please contact the corresponding author.
